# Hyaluronic Acid Dermal Filler‐Associated Vascular Occlusion—A Review of Prevention and Management Strategies

**DOI:** 10.1111/jocd.70884

**Published:** 2026-05-05

**Authors:** Stephen Lowe

**Affiliations:** ^1^ MUSE Clinic Sydney Australia

**Keywords:** adverse events, dermal filler, hyaluronic acid, prevention, treatment, vascular occlusion

## Abstract

**Background:**

Significant complications are uncommon with aesthetic hyaluronic acid (HA) dermal fillers, but inadvertent injection of dermal filler into an artery can result in tissue damage, scarring, visual loss, and even stroke. Practitioners must therefore reduce this risk through proactive, preventative techniques. Early recognition of vascular occlusion (VO) is also crucial to minimize subsequent tissue injury.

**Aims:**

To review and summarize evidence‐based preventative measures and first aid management strategies for HA‐associated peripheral cutaneous VO, explicitly excluding ocular and neurological complications.

**Patients/Methods:**

A comprehensive literature search was conducted using the BestBETs methodology to review the preventative measures available for reducing the risks of HA‐associated peripheral VO, with ocular and neurological side effects considered beyond this review's scope. We then examined the pathophysiology of VO and the evidence for its management to establish an HA dermal filler VO “first aid” protocol.

**Results:**

Our review underscored the importance of preventative strategies (practitioner skill and knowledge; cannula usage; microboluses of small filler volumes, low plunger pressure, and constant needle tip movement), along with the liberal usage of hyaluronidase, heat, and massage, and supports the addition of antiplatelet agents for acute management. Aspiration is controversial and cannot reliably exclude intravascular needle placement.

**Conclusions:**

Prompt recognition and management of VO are critical to prevent skin necrosis, scarring, and long‐term morbidity. Preventative practice, immediate treatment protocols, and further research are essential to enhance clinician confidence and improve patient safety in aesthetic HA filler procedures.

## 1 Introduction

Hyaluronic acid (HA) dermal fillers comprise over 30% of nonsurgical cosmetic procedures worldwide [1, 2]. They create mechanical, volumetric changes in surrounding tissues and can also modulate skin biology [3, 4]. To prolong its half‐life, exogenous HA is typically crosslinked and is generally well tolerated [5, 6]. Variations in cross‐linking, HA concentration and molecular weight generate fillers with different physicomechanical properties and resistance to shear and compression [7], which in turn influence performance in routine use and during adverse events (AEs). HA fillers are typically associated with mild, easily‐managed complications, but vascular AEs can be serious and cause permanent scarring, disfigurement or visual loss and stroke [8]. AEs can arise from the product, injection technique, or adverse inflammatory responses [5]. Complications depend on the product's reversibility as exogenous hyaluronidase can catabolize HA.

Inadvertent filler injection into a blood vessel can cause vascular occlusion (VO) leading to hypoperfusion, distal tissue hypoxia, skin necrosis, blindness, and stroke. Although filler‐associated VO affects 0.015% of patients [9], this may be underestimated, as VO may be mild, misdiagnosed, undiagnosed, or not tracked. Intraluminal filler may partially or completely obstruct vascular perfusion due to inadvertent direct cannulation. Indirect extraluminal obstruction can occur when the vessel is completely perforated and the filler reverses along the injection path into the vessel [10]. External pressure by fillers can compress adjacent vessels, causing occlusions [11]. The clinical consequence of intravascular HA dermal filler depends on the vessel location, filler volume and rheology, and intravascular injection pressures [10, 12]. Intravenous placement may be less frequent than intra‐arterial placement, but can cause thrombophlebitis, pulmonary embolism, and cerebral sinus thrombosis [13]. Depending on the injected volume, viscosity and cohesivity, intra‐arterial filler may enter the circulation, with smaller globules lodging in narrower vessels or becoming resolved when catabolized by endogenous hyaluronidase. Conversely, a large bolus can completely obstruct arterial flow or undergo antegrade or retrograde migration to obstruct surrounding branches [10, 13], extending the impacted area.

Anastomotic vessels [14] allow blood to circumvent a VO, but “choke vessels” spasm upon exposure to intravascular HA [15, 16], limiting collateral blood flow and aggravating hypoperfusion, ischemia and obstructive “red thrombus” formation [17, 18]. Tissue tolerance to ischemia varies, with neuronal and retinal tissue becoming permanently damaged within 15 min [19], while skin tolerates more prolonged ischemia before necrosis, scarring, and disfigurement [20]. However, reperfusion, either by dispersing HA plugs or shunting of blood around it via collateral supplies, can cause ischemia–reperfusion injury. Moreover, inflammatory mediators [21] can further damage endothelial linings by stimulating leukocyte migration and endothelial attachment, further narrowing and vasoconstricting vessels [21].

Rather than providing a treatment algorithm for retinal or other ocular or neurological embolic events, this review addresses peripheral cutaneous VO management only. Retinal or other ocular or neurological embolic events causing visual loss or stroke require distinct, urgent ophthalmology or neurology‐led pathways beyond the scope of this review and proposed first‐aid protocol. Due to limited high‐quality evidence, we used BestBETs methodology to review VO risk reduction, early recognition, and treatment, developing a practical first‐aid algorithm for cutaneous events.

## 2 Materials and Methods

### 2.1 Best Evidence Topics (BestBETs) Reviews

BestBETs uses a simplified, stepwise approach [22] for literature searching and presentation of best‐available evidence in a patient‐focused and clinically‐relevant format [23] for screening of HA dermal filler‐associated VO prevention and management strategies. Briefly, a clinical scenario was presented as a three‐part question following the (P) Patient group (I) Intervention with/without (C) Comparison group and (O) Outcome (PICO) framework [24, 25]. PICO search terms were expanded using Boolean operators (‘OR’ and ‘AND’) for a sufficiently sensitive search [26]. Searches in medical databases (e.g., PubMed) were prioritized, but expanded to others depending on search success, or narrowed with the LIMIT command until a reasonable number of articles were identified. Abstracts were screened for relevance, and selected full‐text papers were critically appraised for major methodological flaws that impacted their validity. Publications with the highest evidence level were prioritized. Data from selected papers were tabulated to highlight key study outcomes and weaknesses. Papers were further analyzed for clinical conclusions that answered the clinical question in the clinical scenario. Where available, we prioritized conclusions supported by systematic reviews and larger case series (CEBM Level 3), and we clearly identified recommendations that were based primarily on expert consensus (CEBM Level 5). Consistent with BestBETs methodology, this approach prioritizes timeliness and clinical applicability over exhaustive retrieval and formal meta‐analysis.

### 2.2 Clinical Scenario Generation

A clinical scenario was described from daily aesthetic practice to ensure a practical, applicable, and clinically‐focused enquiry (Table 1).

**TABLE 1:** Sample Clinical Scenario for BestBETs.

Clinical scenario	
Patient	A 55‐year‐old Fitzpatrick V Aboriginal Australian female presents with long‐term concerns of her nasolabial folds. She has received prior treatment with HA dermal filler to her medial cheek, lateral zygoma, temple region and preauricular zone, to support the lateral face and mitigate the nasolabial fat pad descent. She has no signs or symptoms suggestive of body dysmorphic disorder.
Past medical history	Well. Postmenopausal.
Drug history	No regular medication. No known drug allergies.
Social history	Nonsmoker. Occasional alcohol. Works full time in a bank.
Treatment	HA dermal filler injection (2 mL total): 0.5 mL to each canine fossa and 0.5 mL to each nasolabial fold, administered with sharp needle and cannula
Adverse events/Complications	The patient reports the procedure was more uncomfortable than usual, especially on the left and suspects a bruise due to a purplish discolouration to the left upper nasolabial fold. After 6 h, the patient calls the clinic to report increasing pain on the left side. On review, the purplish zone of discolouration (consistent with livedo reticularis) has extended to the left nasolabial fold, left medial cheek and left lower nose. The capillary refill time is approximately 5 s, and the skin feels cool. The patient is diagnosed with a left angular artery VO from her recent HA dermal filler. She is informed of the clinical diagnosis and the need for further management. She asks what the next step of treatment is and how this could have been avoided.

Abbreviations: HA, hyaluronic acid; VO, vascular occlusion.

### 2.3 Evidence Searching

From the clinical scenario, a three‐part question was formulated incorporating the patient group, intervention, and outcome: What are the [*prevention and treatment*] strategies for [*HA dermal filler*] associated [*VO*]? This question was expanded to ensure sufficiently specific but broad search terms to capture relevant publications (Table 2). Inclusion criteria were full‐text systematic reviews or reviews published within the last 10 years in the English language. PubMed was searched first, followed by The Cochrane Library, Embase, and Scopus for thoroughness, using specific terms adjusted based on suggestions from each search engine and to capture all important papers (searches concluded 1 June 2023). Initially, the terms (Appendix 1) included retinal VO and non‐HA dermal fillers (calcium hydroxylapatite, poly L‐lactic acid, and polycaprolactone), but the results were too extensive and fell outside our predefined focus on HA‐associated peripheral cutaneous VO. These expanded terms were therefore excluded, and the search narrowed to “HA dermal fillers” and “peripheral VO”, in line with our exclusion of ocular and neurological complications from the treatment algorithm. References within the papers were screened for commonly quoted publications and included if relevant and could directly assist in clinical scenario responses, even if they had lower levels of evidence. Exclusion criteria were non‐English publications, articles older than 10 years, and nonfull‐text sources.

**TABLE 2:** BestBETs PI (C)O search terms.

PICO term	Element	Search terms
Patient/Population (P)	Patients receiving hyaluronic acid fillers	Adults undergoing aesthetic treatments
	Immediate hyaluronidase protocol	
Intervention (I)	Dermal filler injection recipients	Hyaluronidase injection
	High‐dose hyaluronidase treatment	
Comparison (C)	No immediate intervention	Standard supportive care
	Delayed treatment	
	Alternative reversal agents	
Outcome (O)	Prevention of tissue necrosis	Resolution of vascular occlusion
	Time to symptom resolution	

### 2.4 Search Outcome

An initial yield of 633 results was further filtered to 75 results. Abstracts were screened for relevance, followed by full‐text screening and duplicate removal. Systematic reviews were prioritized, but due to their scarcity, reviews were considered. Bibliographies were checked for relevant publications.

## 3 Results

### 3.1 BestBETs Table

**FIGURE 1:**
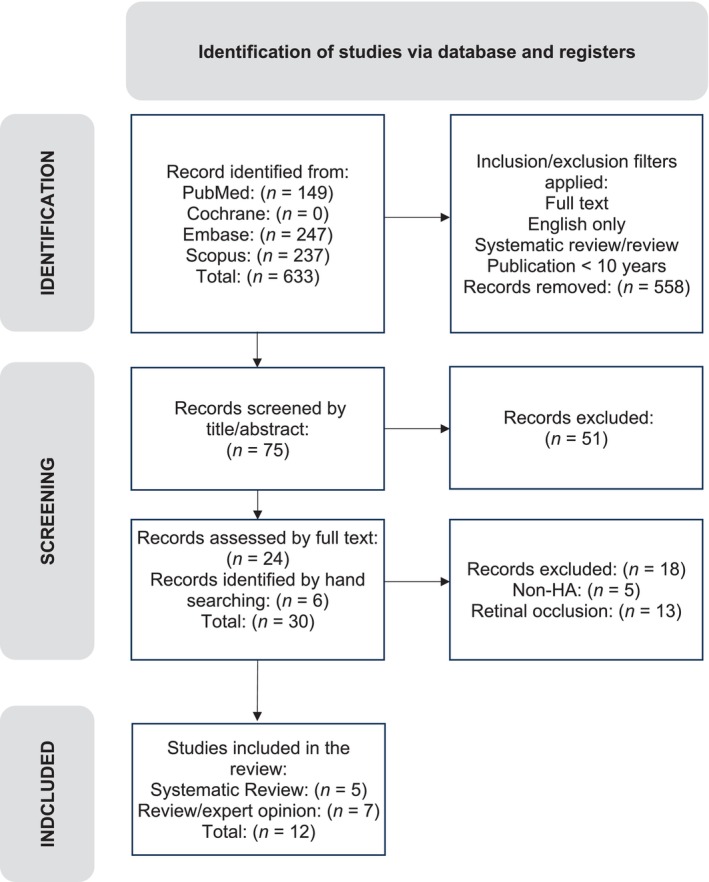
Identification of studies via database and registers.

Twelve papers were included for further appraisal, including five systematic reviews Centre for Evidence Based Medicine CEBM Level 3 and one expert opinion review paper CEBM Level 5. Six additional expert opinion reviews (CEBM Level 5) derived from paper references were added due to their direct clinical relevance (Figure 1). Together, these sources allowed us to differentiate evidence‐based recommendations grounded in systematic reviews and larger case series from practice recommendations relying predominantly on expert consensus. All papers were tabulated according to BestBETs methodology (Table 3) for analysis of their methodological strengths and weaknesses and to establish their validity.

**TABLE 3:** BestBETs table.

Author, date, and country	Patient group	Study type and CEBM level of evidence	Outcomes	Key results	Study weaknesses/comments
Aviv U 2020. Israel [27].	52 case studies/series HA related vascular occlusion 107 patients (98 female/9 male) mainly from Asian countries Mean age: 35.26y	Systematic review Level of Evidence: 3a	Areas injected Skin vs. ocular symptoms Time to initial treatment Hyaluronidase dosage, number of treatments and injection technique Additional treatments Outcome	Management of skin necrosis and visual disturbance variable with no established consistent approach Time to intervention is of high importance to halt progression of tissue damage	Small sample sizes from each study All cases are retrospective case studies Exclusion of non‐English papers No risk of bias assessment in included papers
Al‐Alam Sansur S. 2022. Palestine [28]	72 studies with 186 patient cases Case reports = 56 Case series = 10 Retrospective studies = 6 Mean age: 37 years 8 females, 8 males	Systematic review Level of Evidence: 3a	Timing of treatment initiation Type of intervention used	Predictable and satisfactory outcomes with immediate high‐dose hyaluronidase Treatment within 24 h halted progression of necrosis	Lack of high level of evidence (case reports/case series) Lack of prospective studies Multiple treatment modalities confound outcome results
Nayfeh T 2021 USA [29]	182 studies Some included studies had huge patient numbers (> 7 million patients)	Systematic reviewEvidence: 3a—	Incidence and risk factors of vascular occlusion Treatment of VO Incidence and risk factors and treatment of nodules and inflammatory events related to HA and non‐HA fillers	HA used > 80%, CaHA ~10%, PLLA ~5% Skin necrosis approx. 5/1000 but wide reported rates between studies Hyaluronidase key treatment, variable dose range 150‐1500iu, median time to treatment 45 h Multiple secondary treatments discussed 77% of cases shown improvement	Collection of small, heterozygous studies Lack of comparative studies
Jones D 2021 USA [30]	182 studies Some included studies had huge patient numbers (> 7million patients)	Systematic review and guidelines accompanying Nayfeh T [29]Evidence: 3a—	Prevention of VO, blindness and stroke Treatment of filler‐related VO with blindness Treatment of filler‐related VO without blindness (skin ischaemia) Reducing and treating nodules from HA filler/permanent and semi‐ permanent fillers	Anatomy knowledge; injector knowledge; cannula preference; low plunger pressure; consent. Hyaluronidase is key	Collection of small, heterozygous studies Lack of comparative studies
Sito G 2019 Italy [31]	30 articles 93 cases F = 84 M = 9 Mainly China/Korea	Systematic review with meta‐analysisLevel of Evidence: 3a—	Injected substance Injection site Affected blood vessel Main consequence Concomitant symptoms Imaging tests Time to symptom onset Outcome	Blindness main consequence (57/93 = 61%) Partial/total recovery (24/93 = 28%) No improvement (61/93 = 72%) HA and autologous fat most commonly implicated	Weak methodology with lots of bias Incomplete data Study heterogeneity
King M 2020 UK [32]	No patient group	Exert opinion, review and guideline Level of Evidence: 5	Incidence Signs and symptoms of VO Areas of caution Minimizing VO risk Treatment of VO	Comprehensive summary and practical management algorithm	No methodological description No critical evaluation Lower level of evidence
Murray G 2021 UK [33]	No patient group	Expert opinion, review and guideline Level of Evidence: 5	Pharmacology of hyaluronidase Indications	Comprehensive summary Intradermal testing guidelines Algorithm of modified high‐dose pulsed protocol	No methodological description No critical evaluation Lower level of evidence
DeLorenzi C 2013 Canada [7]	No patient group	Expert opinion and review Level of Evidence: 5	Pathology and pathophysiology of VO Risk factors Treatment	Signs and symptoms of VO Risk factors for VO: site, volume, needle, scarring, cannula, filler type Treatment: “filler crash kits”	No methodological description No critical evaluation Lower level of evidence
Murray G 2021 UK [34]	No patient group	Expert opinion and review Level of Evidence: 5	Causes of VO Mitigating risk of VO Assessing a VO Management	Establishing a clinical diagnosis Targeted therapy with hyaluronidase	No methodological description No critical evaluation Lower level of evidence
Signorini M 2016 Italy [35]	No patient group	Consensus expert opinions Level of Evidence: 5	Vascular compromise Inflammatory reactions Injection related Product related	Strategies to minimize risk of complications VO treatment algorithm	Lower level of evidence No critical evaluation
Goodman G 2020 Australia [3]	No patient group	Consensus expert opinions Level of Evidence: 5	Patient consent process Preventative strategies High risk areas Role for hyaluronidase and anti‐coagulation	Consent patients adequate for rare but significant risks Risk reduction strategies: anatomy, cannulas, slow injection and low pressure, microboluses. No evidence to support aspiration Higher vs. lower risk zones Avoid anti‐coagulation for retinal occlusion	Lower level of evidence No critical evaluation The study focus was retinal occlusion, but the principles relate to all vascular occlusions.
DeLorenzi C 2017 Canada [6]	No patient group	Consensus expert opinions Level of Evidence: 5	Resolution of HA related vascular occlusion Formation of updated hyaluronidase treatment protocol	High Dose Pulsed Hyaluronidase reportedly more effective and simpler than prior protocols Favorable outcomes without relying on ancillary treatments of unproven benefit	Lower level of evidence No RCT to support, experiential reports only

### 3.2 Methodological Evaluation

Papers were critically reviewed for validity of their conclusions. One systematic review [27] used adequate search terms, reputable source paper reference libraries, and strict exclusion criteria to collect more homogeneous studies, although relevant publications may have been omitted. The small sample sizes of the included papers were an acknowledged weakness and affected most studies included. Although two authors screened the papers for inclusion, they were not assessed for methodological quality or bias.

Another systematic review [28] had a robust clinical focus, a Preferred Reporting Items for Systematic Reviews and Meta‐Analyses (PRISMA) study design [29], robust inclusion/exclusion criteria, and a thorough search strategy including multiple reviewers and hand‐searching of relevant references. Two reviewers evaluated studies using critical appraisal tools for risk of bias and completeness of reporting.

Two related, high‐quality systematic reviews [30, 31] followed PRISMA statements [29] and included medical librarian assistance to minimize selection bias. Inclusion and exclusion criteria were strict and comprehensive, and appraisals and disagreements were conducted independently. Bias was assessed using an adapted Newcastle‐Ottawa scale for observational studies [32], the Cochrane risk of bias tool for randomized controlled trials (RCTs) [33], and uncontrolled studies were also assessed [34]. Study heterogeneity was evaluated using *I* [2] and Cochrane Q tests. Study weaknesses such as incomplete data, lack of comparators, and the presence of small, heterogeneous studies were identified.

One systematic review [35] lacked methodological rigor, with less‐comprehensive searches, ambiguous inclusion and exclusion criteria, and no references to assessments of methodological quality. Publication bias was evident, with inclusion of papers by acquaintances and overrepresentation of case reports documenting blindness, indicating overestimations of visual loss. Most of the included papers were from Korea and China, affecting their generalisability. The heterogeneity of the included studies, although acknowledged, undermined the meta‐analysis reliability.

The remaining seven studies were expert opinion reviews (CEBM Level 5) [12, 13, 36, 37, 38, 39, 40], included due to their direct relevance to the original clinical question. Although of lower evidence, all papers contribute valuable experience and expert opinions to the subject of VO prevention and management. Their inclusion helps derive academically sound, practical, and relevant conclusions to shape recommendations for improved clinical practice.

## 4 Discussion

HA filler‐associated VO can cause substantial morbidity and patient distress. We examined the evidence for VO prevention and best‐practice management strategies, focusing on peripheral VO from HA dermal fillers. Using a BestBETs search and a clinical scenario of a patient who developed postfiller VO, we identified twelve CEBM Level 3 and 5 papers, reflecting the reality that aesthetic medicine relies heavily on small series and expert opinions. Where possible, our prevention and management recommendations are anchored in systematic reviews and larger case series (CEBM Level 3). Where only lower‐level data were available, we present expert consensus as pragmatic guidance rather than definitive, evidence‐based standards of care. Our findings are presented as ‘prevention’ or ‘management’ strategies to enhance safety and effectiveness.

### 4.1 Prevention

#### 4.1.1 Anatomy Knowledge and Injector Skill

Aesthetic medicine is inadequately regulated in many countries, resulting in inconsistent training standards and qualifying requirements. Aesthetic medicine would be improved by its recognition as a specialty, enforcement of robust training, accreditation, continual professional development standards, and fellowship examinations by training colleges. Manufacturers must be involved as they provide training but currently set their own training standards without regulatory requirements of minimum standards for injectors.

Inexperience [39] contributes substantially to complications. Thorough knowledge of three‐dimensional, injection‐related anatomy and vascularity [41] together with proper injection techniques is therefore essential [35, 38]. Injecting areas around bony foramina should be avoided, while scar tissue from prior surgery or repeated filler treatments [13] that may increase intravascular cannulations should be identified. Facial vascular anatomy should be studied [31] through cadaveric dissection and awareness of anatomical variations. Others [40] advocated identifying higher and lower (jawline and chin) vascular risk facial zones and treating medium (cheeks and lips) and higher risk zones (glabella, nose, forehead, temples, nasolabial fold, and tear troughs) only by clinically experienced injectors [41]. However, such enforcement requires regulators, training colleges, and indemnity insurance providers. Doppler ultrasound for preprocedural arterial mapping and real‐time guidance represents an emerging prevention strategy, particularly valuable for identifying vascular variations in high‐risk zones [41].

#### 4.1.2 Needle Versus Cannula

Needles and cannulas can effectively deliver dermal fillers, but no single injection technique is entirely safe, and operator knowledge and technique mastery are important [39]. Cannulas can cause VOs and enter arteries (except 10G cannulas) [42]. Larger cannulas (≥ 25G) require greater force for vessel entry than needles. Five papers [13, 31, 36, 38, 40] recommended larger cannulas (≥ 25G) to reduce injection risks, which the American Society for Dermatologic Surgery (ASDS) rated as a Strong Recommendation with Moderate certainty evidence as per GRADE recommendations [43]. However, recent reports of blindness following nasal injections despite cannula use emphasize that technique (micro‐aliquots ≤ 0.025 mL, low pressure, constant movement, perpendicular vessel approach) remains paramount regardless of instrument [44]. Differences in cannula lengths, tip shapes, and tip sharpness may impact their blood vessel penetrability [44]. Cannulas should not be injected parallel to major vessels to minimize inadvertent cannulation; significant vessels should be approached perpendicularly [40]. Cannula aspiration to check for intravascular presence may be mechanically challenging due to the cannula lengths, higher negative pressures, and aspiration times [44].

#### 4.1.3 Injected Product Volume and Injecting Pressure

Injecting small volumes (up to 0.1 mL/injection) [13, 30, 31, 35, 36, 38, 39, 40] prevents larger arterial obstructions, while maintaining needle or cannula movement minimizes prolonged intravascular injections. In higher‐risk zones (e.g., nose), injections should be limited to microboluses (< 0.025 mL) [36, 45, 46]. As the average volume of the supratrochlear artery from the glabella to the orbital apex is 0.085 mL (range 0.04–0.12 mL) [47], microbolus injections lessen retinal VO risks. Slow injections with low plunger pressures were also advocated [12, 13, 30, 31, 35, 36, 38, 39, 40], but depend on syringe design and filler rheology [7]. Gentle, low‐pressure injections reduce inadvertent widespread intravascular filler dissemination by not exceeding mean arterial pressures, limiting subsequent retrograde filler migration. Smaller, narrower needles and syringes deliver precise aliquots [39] slowly, but clogs require increased pressure to resolve. Higher plunger pressures may worsen intravascular plugs [13, 45], which are more likely with higher‐viscosity and higher‐cohesivity HA fillers than lower‐viscosity and lower‐cohesivity fillers [10, 13, 48].

#### 4.1.4 Aspiration

Performing preinjection aspiration is clinically valuable when blood flashback occurs upon syringe retraction and prompts repositioning prior to injection. However, viscous filler within the cannula or needle lumen can hinder aspiration, creating false negatives and a misleading sense of safety [31, 35, 39, 49]. The ASDS rated aspiration as a “best practice” with a Strong Recommendation and moderate/high certainty, primarily as a pragmatic safety step within expert consensus guidelines. However, prior publications have demonstrated a highly variable true‐positive rate and substantial dependence on needle diameter and length, filler rheology, and the amount and duration of negative pressure applied to the syringe, making aspiration an unreliable test for intravascular placement [49, 50]. There remains ambivalence about aspiration [13, 36, 38], a need for awareness of its limitations, and not relying on negative aspiration. One paper [40] recommended more strongly against aspiration as it was unreliable, variable, may encourage unsafe practices, and involved excessive needle/cannula tip movement which compromised aspiration [51]. It recommended constant needle or cannula oscillation [40] to minimize intravascular placement and overall VO risks. Aspiration can therefore be considered as an adjunctive maneuver within expert consensus algorithms, but a negative aspiration result should not be interpreted as evidence‐based confirmation of safety [30, 31, 49, 50].

#### 4.1.5 Additional Measures

Adrenalised local anesthetic induces local vasoconstriction but can be confused for occlusion‐induced pallor. Patients should consent to all possible risks associated with elective cosmetic procedures and freely decide on their acceptable level of risk.

### 4.2 Management

#### 4.2.1 Clinical Presentation

**FIGURE 2:**
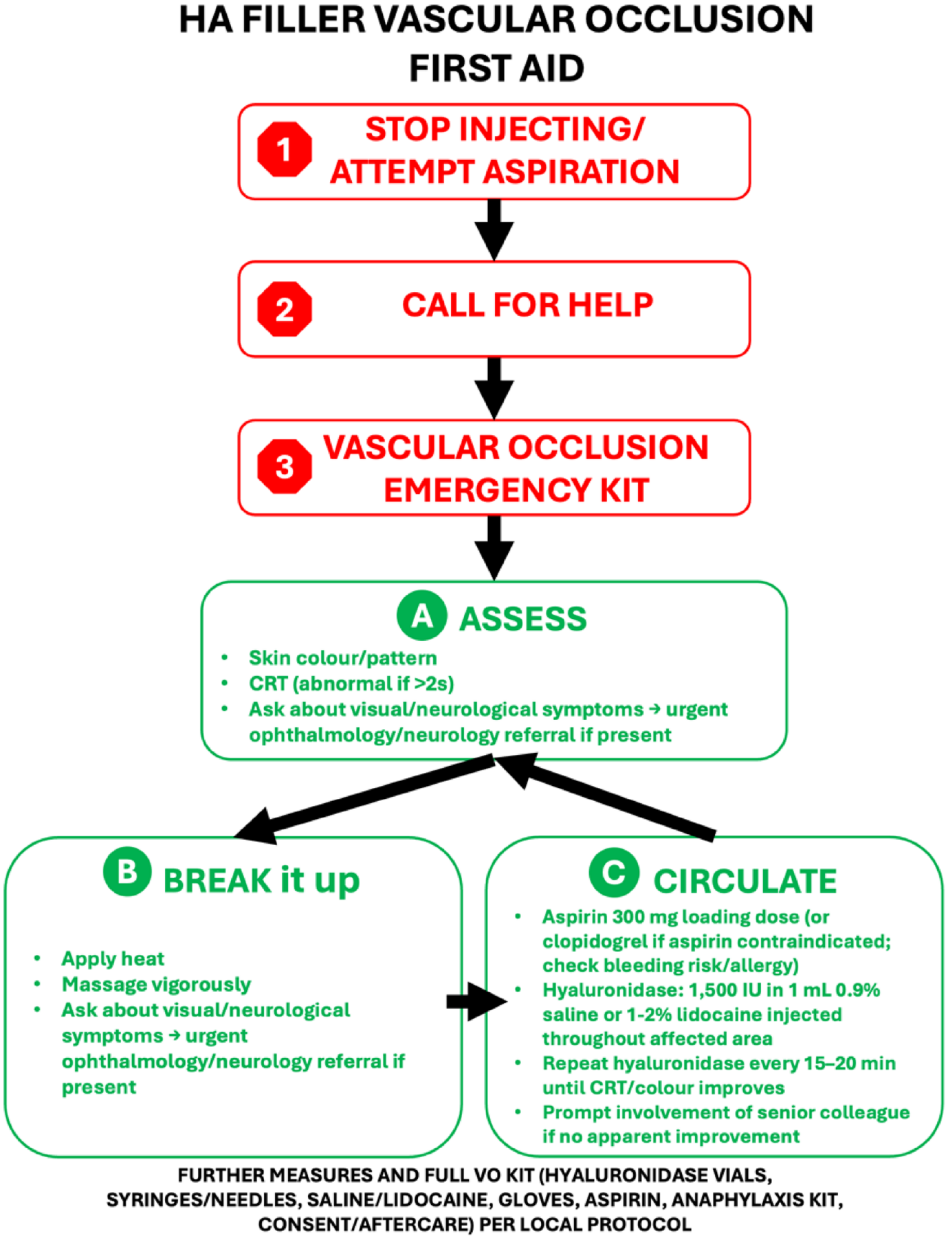
HA dermal filler First Aid emergency protocol for peripheral cutaneous vascular occlusion (not ocular or neurological complications).

Arterial HA filler occlusion affecting the skin and subcutaneous tissues requires prompt recognition, diagnosis, and treatment. VO can be evident immediately at injection, or much later [12]. This Management section and the associated First Aid protocol (Figure 2) are intended for peripheral cutaneous VO only and are not designed to manage retinal, cerebral or other neurological embolic events. Blanching develops from a sudden, filler plug‐associated hypoperfusion, and may be temporary and easily missed or attributed to concomitant topical anesthesia or adrenalised injected local anesthetic. Blanching is replaced by a reticular, dusky purple color (livedo reticularis [52]) as deoxygenated blood accumulates post‐VO. Livedo reticularis can be mistaken as bruising but results from intravascular obstructions such as HA filler‐associated occlusion and other emboli [52], expanding with increasing hypoxia [12, 13]. Recent HA filler injections suggest a livedo reticularis diagnosis, but its detection can be challenging on skin of color or under local anesthesia. Pain postprocedure and prolonged capillary refill time (CRT) (> 2 s) suggests hypoperfusion and may indicate VO [13]. Livedo reticularis and reduced CRT do not indicate inevitable skin necrosis, as some tissues can tolerate ischemia [20], but persistent VO necrotizes skin. The extent of tissue damage depends on the occlusive plug size, success of collateral blood supply, underlying patient health, and presence of secondary infections [38]. Persisting skin ischemia causes coagulative necrosis, progressive cell lysis, tissue darkening, and inflammatory cascades. Good wound care protects the surrounding skin, promotes healing and minimizes secondary infections but prolonged repair can cause scarring [13, 36, 38]. Paradoxically, when vascular supply is reestablished, reperfusion injury can aggravate wound healing, causing further tissue injury [21].

#### 4.2.2 Emergency Management

Figure 2 provides an evidence‐based “First aid” protocol for the initial emergency management of HA‐associated VO. Dermal filler aspiration could be attempted if the needle/cannula remains in situ. Subsequent management steps should be expedited [36]. The area can be massaged and heat applied to mechanically stimulate filler disruption and local vasodilation [13, 36, 38]. Failure of conservative measures to promptly reestablish normal CRT and skin perfusion may require hyaluronidase.

#### 4.2.3 Hyaluronidase

Hyaluronidase catalyzes HA hydrolysis of glycosaminoglycan polysaccharides [37]. It treats visible or cosmetically unflattering filler, or HA‐related delayed‐onset nodules. In emergencies, higher concentrations dissolve VO‐associated HA plugs.

BestBETs analysis identified hyaluronidase as the gold standard for VO management [12, 13, 27, 28, 30, 31, 35, 36, 37, 38, 39, 40] In Australia, Hyalase (1500 IU/vial) is available; practitioners should consult local Product Information for dosing [37]. High‐dose, concentrated volumes administered regularly were favored until resolution [13, 28, 30, 31, 36, 37, 38]. One protocol administers 500 units per zone (half upper lip area) hourly into the compromised area [5, 12, 13]. Another author recommends an initial loading dose of 1500 IU in 1 mL of 0.9% saline or 1%–2% lidocaine infiltrated along artery and compromised areas, repeated every 15–20 min until CRT normalizes [37, 38]. One systematic review [28] considered these high‐dose pulsed protocols to be predictable, satisfactory, and able to prevent necrotic progression when initiated within 24 h of occlusion. The ASDS also strongly recommends this high‐dose pulsed hyaluronidase approach [31], High concentrations in low‐volume diluents minimize diffusion from VO site. Subcutaneous injection suffices as hyaluronidase crosses fascial planes [12]. Perfusion restoration should be prioritized over exact units, while preadministration allergy testing is unnecessary given low hyaluronidase hypersensitivity risks [37], though anaphylaxis equipment must remain accessible. Accurate medical notes should be recorded, including cumulative drug doses, to ensure safe dosages are not exceeded (i.e., lidocaine). Ultrasound guidance for hyaluronidase injections, though not captured in the primary literature search, warrants mention as recent reports demonstrate improved localization of intravascular filler and targeted enzyme delivery [9].

#### 4.2.4 Anti‐Platelet Agents

A single antiplatelet agent (aspirin or clopidogrel, selected based on patient contraindications, bleeding risk and local protocols) can limit platelet aggregation around intra‐arterial HA plugs [17, 18]. A typical regimen involves a 300 mg loading dose followed by 75 mg daily until reperfusion [13, 27, 28, 36, 38]. Anti‐platelet agents can commence prehyaluronidase to minimize early platelet aggregation [27], though these off‐label recommendations rest on low‐quality evidence from case series and expert consensus. Accordingly, their use should be individualized and framed explicitly as expert consensus rather than evidence‐based standard of care.

#### 4.2.5 Other Agents

Evidence for the routine administration and therapeutic benefits of other drugs is lacking. Nitroglycerin paste showed no benefit in vasodilation and may worsen compromise [53] and can cause protective choke vessel dilation and release of the HA embolus into adjacent angiosomes [15]. Thrombus reduction with low molecular weight heparin lacks adequate evidence as a standard treatment and should only be considered if anti‐platelet agents are contraindicated [54]. Phosphodiesterase‐5 inhibitors [36, 38], prostaglandin E1 analogues, pentoxifylline, hyperbaric oxygen therapy and steroids should be used on a case‐by‐case basis as recommendations for their use are derived from expert opinion and low‐level data and cannot be regarded as standard, evidence‐based therapy [13, 28, 36]. Inaccessibility to treatments such as hyperbaric oxygen therapy may prevent their routine use. Antibiotics and anti‐virals should be used as indicated or if the risk of wound superinfection is high.

#### 4.2.6 Wound Care

Chronic care of necrotic wounds is essential in the ongoing management of established VOs and depends on wound severity, location and underlying patient health. Plastic surgery and tissue viability specialist nursing staff assistance may be required.

### 4.3 Limitations of BestBETs

We were limited by having only one reviewer during publication searches, appraisals, and selection [55, 56], causing some selection bias. Moreover, we excluded non‐English papers, those > 10 years old, and gray literature. BestBETs provides pragmatic evidence summaries, not comprehensive systematic reviews with formal bias assessment or meta‐analysis. Conclusions represent evidence‐informed guidance, not definitive standards. Searches concluded 1 June 2023 and subsequent publications have not yielded high‐level evidence altering these consensus recommendations.

### 4.4 Novel Strategies

Emerging prevention strategies include routine Doppler ultrasound vascular mapping and refined postcare protocols, while prospective registries will strengthen the evidence base beyond current expert consensus. These future developments should ideally be integrated with separate, specialty‐led pathways for ocular and neurological complications, which remain beyond the scope of this review.

## 5 Conclusion

While HA dermal fillers provide great aesthetic benefits, the management of VO risks and incidents requires interventions to be implemented promptly, judiciously, and in combinations. Core VO management strategies form the basis of the proposed Vascular Occlusion First Aid protocol, while other treatments with a poor evidence base can be considered as second‐line treatments. Practitioners should undertake training in the early recognition, diagnosis, and first aid management of cosmetic emergencies, or be encouraged to use tools such as ultrasound mapping for pretreatment assessments and identification of key anatomical structures and variations.

## Author Contributions

The author contributed entirely to conceptualization, review synthesis, and manuscript approval, adhering to COPE guidelines for publication ethics.

## Funding

Funding for manuscript editing and preparation was provided by Merz Asia Pacific Pte Ltd. to Dr. Shawna Tan, Medical Writers Asia.

## Ethics Statement

The author has nothing to report.

## Consent

The author has nothing to report.

## Conflicts of Interest

Stephen Lowe is a consultant and speaker for Merz Aesthetics and Sciton.

## Data Availability

The data that support the findings of this study are available from the corresponding author upon reasonable request.

## Appendix 1 Terms Used for Online Literature Search in PubMed, The Cochrane Library, Embase and Scopus

**Table:**

Online literature search		
Database	Terms	Outcomes
PubMed (via Medline): searched 01/06/2023	((((“injectable filler”) OR (“dermal filler”) OR (“hyaluronic acid”)) AND ((((prevention) OR (treatment)) OR (risk)) OR (management))) AND ((“vascular occlusion”) OR (“skin necrosis”))	Initial outcome: 149 search items Filters applied: Full text, review, systematic review, English, 10 years Outcome: 23 search items
The Cochrane Library: searched 01/06/2023	“Dermal filler” AND “complications”	Outcome: 0 search items
Embase: searched 01/06/2023	‘skin necrosis’ OR ‘blood vessel occlusion’ AND ‘hyaluronic acid’ OR ‘injectable dermal implant’ AND ‘risk’ OR ‘management’ OR ‘treatment’ OR ‘prevention’	Initial outcome: 247 search items Filters applied: 2013–2023, systematic review Outcome: 9 search items
Scopus: searched 01/06/2023	“skin necrosis” OR “vascular occlusion” AND “hyaluronic acid” OR “dermal filler” OR “injectable filler” AND “risk” OR “management” OR “treatment” OR “prevention”	Initial outcome: 237 search items Filters applied: 2013–2023, medicine, reviews, English Outcome: 43 search items
